# Back to the bones: do muscle area assessment techniques predict functional evolution across a macroevolutionary radiation?

**DOI:** 10.1098/rsif.2021.0324

**Published:** 2021-07-21

**Authors:** Karl T. Bates, Linjie Wang, Matthew Dempsey, Sarah Broyde, Michael J. Fagan, Philip G. Cox

**Affiliations:** ^1^ Department of Musculoskeletal Biology, Institute of Life Course and Medical Sciences, University of Liverpool, The William Henry Duncan Building, 6 West Derby Street, Liverpool L7 8TX, UK; ^2^ Department of Engineering, University of Hull, Hull HU6 7RX, UK; ^3^ Department of Archaeology, University of York, PalaeoHub, Wentworth Way, Heslington, York YO10 5DD, UK; ^4^ Hull York Medical School, University of York, Heslington, York YO10 5DD, UK

**Keywords:** macroevolution, biomechanics, multi-body dynamics, finite-element analysis, rodent mastication

## Abstract

Measures of attachment or accommodation area on the skeleton are a popular means of rapidly generating estimates of muscle proportions and functional performance for use in large-scale macroevolutionary studies. Herein, we provide the first evaluation of the accuracy of these muscle area assessment (MAA) techniques for estimating muscle proportions, force outputs and bone loading in a comparative macroevolutionary context using the rodent masticatory system as a case study. We find that MAA approaches perform poorly, yielding large absolute errors in muscle properties, bite force and particularly bone stress. Perhaps more fundamentally, these methods regularly fail to correctly capture many qualitative differences between rodent morphotypes, particularly in stress patterns in finite-element models. Our findings cast doubts on the validity of these approaches as means to provide input data for biomechanical models applied to understand functional transitions in the fossil record, and perhaps even in taxon-rich statistical models that examine broad-scale macroevolutionary patterns. We suggest that future work should go back to the bones to test if correlations between attachment area and muscle size within homologous muscles across a large number of species yield strong predictive relationships that could be used to deliver more accurate predictions for macroevolutionary and functional studies.

## 1. Introduction

Calculations of the force-generating capacity of muscles, based on measurements of muscle attachment sites and/or areas delineated by osteological structures, are widely used in macroevolutionary studies of functional morphology and biomechanics (e.g. [1–27]). These muscle area assessment (MAA) techniques have been applied to limbs (e.g. [22–24]) and the axial skeleton (e.g. [25–27]) but are most frequently used in skulls (originating from the ‘dry skull method’ [1]) to examine masticatory evolution in both extinct and extant taxa (e.g. [1–21]). For extinct taxa, they provide a means to derive quantitative estimates of muscle proportions, force output and bone loading based on fossilized osteology alone, thereby circumventing the absence of muscle itself in the fossil record. In extant taxa, extrapolating muscle size and mechanical performance from existing bony specimens circumvents time-, labour- and skill-intensive physiological and biomechanical experiments on live animals and/or cadavers, making it feasible to analyse large sample sizes statistically and rapidly, and thus assess broad-scale macroevolutionary patterns (e.g. [2–4,10,12,21]). Although rarely discussed explicitly as a benefit, this also minimizes the need to expose animals to experimentation and euthanasia, thus adhering to the principles of the 3Rs (Replacement, Reduction and Refinement) in scientific research [28], assuming model predictions are accurate enough to satisfy research goals.

However, the ability of MAA-based methods to accurately reconstruct qualitative and quantitative functional patterns in a macroevolutionary radiation has not been extensively tested. To date, measures of accuracy have largely been restricted to single taxon studies of muscle anatomy and bite force [1,29–34]. The varying levels of inaccuracy recovered by these studies contrasts somewhat with a single comparative study of bats, which found that the method accurately predicted bite forces despite inaccurately predicting muscle parameters [35]. In addition to the limited assessment in explicit macroevolutionary contexts, to our knowledge, no study has addressed the absolute or relative inaccuracy that MAA-based methods yield in finite-element studies of bone stress/strain, despite widespread combined use of these approaches. The extent to which MAA reconstruction approaches accurately predict quantitative or even qualitative patterns in macroevolutionary studies is, therefore, poorly constrained.

In this study, we extend a recently published examination of soft tissue reconstruction and biomechanical modelling in macroevolutionary studies [36] to MAA-based approaches to assess quantitatively the capacity of these methods to correctly predict established differences between macroevolutionary morphotypes. This not only allows us to assess the qualitative and quantitative accuracy of MAA-based approaches, but also enables comparisons with alternative volumetric sculpture methods widely used in palaeontological studies (e.g. [36–42]).

## 2. Material and methods

To assess the accuracy of MAA approaches, we used the skeletal, multi-body dynamics analysis (MDA) and finite-element (FE) models of the grey squirrel (*Sciurus carolinensis*), brown rat (*Rattus norvegicus*) and domestic guinea pig (*Cavia porcellus*) presented by Broyde *et al*. [36]. These taxa are representative of masticatory morphotypes within the Rodentia (sciuromorph, myomorph and hystricomorph), and have evolved disparate masticatory musculature and bite mechanics [43–47]. Models of these taxa allowed us to measure the accuracy of MAA approaches for predicting muscle physiological cross-sectional area (PCSA), bite force and bone stress against model iterations that use muscle force-generating properties directly measured through dissection and imaging [46,47]. These models, built using muscle parameters measured in the same specimens being modelled, are referred to here as the ‘extant model’ iterations, as in Broyde *et al*. [36].

Here, we investigated the accuracy of two MAA-based approaches: the dry skull method of Thomason [1], which estimates the summed PCSAs of important muscle groups based on measures of the accommodation space available for these muscles; and a potentially higher-resolution approach in which PCSAs were estimated based on the bony attachment area (AA) of each individual muscle. To measure individual muscle AAs in the models, we used the already defined attachment regions in the FE models (as in [36]; see electronic supplementary material, for more details) and these values were used as the PCSAs for each muscle in the MDA models. For the dry skull model iterations, the temporalis muscle PCSA input into the MDA models was set to the value derived from the MAA for this muscle following Thomason [1], while the PCSA from the masseter + medial pterygoid MAA was divided equally between the posterior line of action of the posterior deep masseter, the anterior line of action of the superficial masseter and the medial pterygoids in the MDA model for each species. All other muscles were removed from the MDA models to reflect the aggregation of muscle PCSA and force output into simplified temporalis and masseter + pterygoid groups by the dry skull method (electronic supplementary material, figure S7). In addition to incisor bite force, we also calculated the mechanical efficiency of bites as the ratio of the bite force to the summed muscle forces, as done previously for these rodents by Cox *et al*. [46]. Predicted muscle forces from MDA models were then also used as inputs in the FE simulations. For the dry skull FE models, muscle forces derived from the masseter+medial pterygoid MAA were divided equally across the attachment sites of all masseter muscles and the medial pterygoids, while the temporalis AA received the temporalis MAA derived force. All other muscle AAs were not loaded, again to reflect the aggregation of muscle forces in the dry skull method. All other parameters remained unaltered from the ‘extant iteration’ of models presented in Broyde *et al*. [36].

## 3. Results

### 3.1. Physiological cross-sectional area

**Figure 1.**
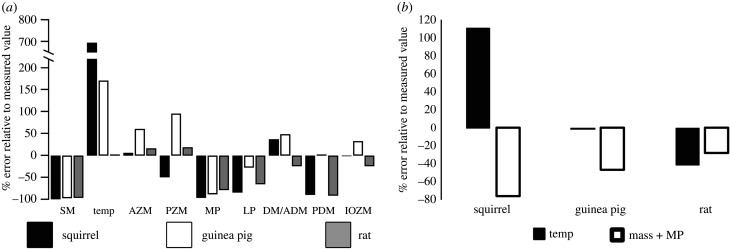
Relative error in PCSA given by (*a*) the AA and (*b*) the dry skull method. Error magnitudes represent the percentage error in the AA and dry skull values relative to the measured PCSA values in the rodent specimens being modelled [43,46,47].

Both MAA approaches varied widely in the accuracy with which they estimated muscle PCSA in the three rodent morphotypes (figure 1*a,b*; electronic supplementary material, tables S1–S4). The AA method gave similar average relative error magnitudes per muscle in the three species (25–40%), but with considerable qualitative and quantitative variation within individual muscles (figure 1*a*; electronic supplementary material, tables S1–S3). In some cases, the AA method gave similar errors in homologous muscles across the three morphotypes: the superficial masseter PCSA was underestimated by 96–99.3% in the three morphotypes; error in the medial pterygoid ranged from −78.2% to −96.3%; and the PCSA of the posterior deep masseter was underestimated by 89% and 91.4% in the squirrel and rat (figure 1*a*; electronic supplementary material, tables S1–S3). However, other muscles varied in both the nature and magnitude of error. For example, the temporalis predictions yielded error of +694.5% and +171% in the squirrel and guinea pig compared to just +2.4% in the rat. The AA method underestimated the PCSA of the posterior zygomatico-mandibularis in the squirrel by 49.5% but overestimated it by 19.3% and 95.8% in the rat and guinea pig (figure 1*a*; electronic supplementary material, tables S1–S3). These errors led to the AA approach correctly ordering taxa in the relative PCSAs of homologous muscles only 10 out of 25 times (40%).

Similar error magnitudes and inconsistencies were recovered for the dry skull method (figure 1*b*; electronic supplementary material, table S4). Temporalis PCSA was overestimated by 110.5% in the squirrel but underestimated by 41.8% in the rat and just 0.2% in the guinea pig (figure 1*b*; electronic supplementary material, table S4). However, the masseter+medial pterygoid predictions all underestimated the real summed PCSAs of these muscles, by 28%, 46.4% and 75.3% in the rat, guinea pig and squirrel. These errors led to the dry skull method correctly ordering taxa in their relative PCSAs in one out of six cases.

### 3.2. Bite force and mechanical efficiency

**Figure 2.**
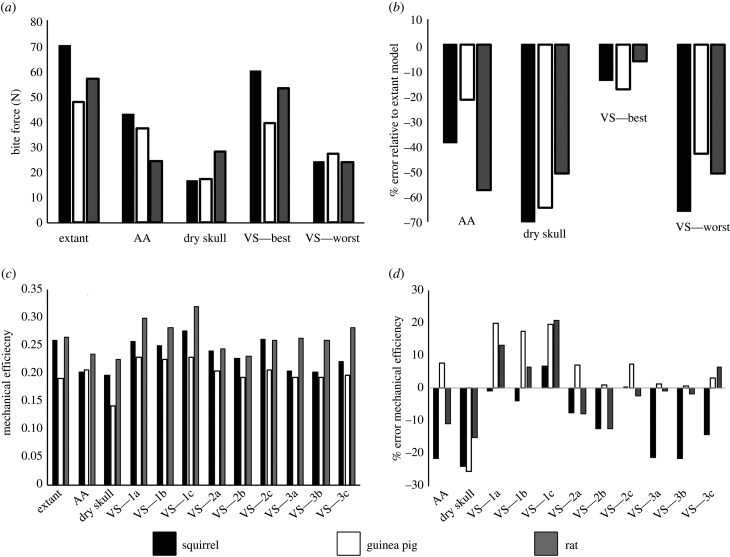
Absolute values and relative error in maximal incisor (*a*,*b*) bite force and (*c*,*d*) mechanical efficiency in MDA models built using PCSAs from the AA and dry skull method compared to extant MDA model iterations, and those generated previously using the volumetric sculpture (VS) approach [36]. Error magnitudes in (*b*,*d*) represent the percentage error in the AA, dry skull and/or volumetric sculpture values relative to the extant MDA model bite force and mechanical efficiency values for each taxon [36,46,47].

When PCSAs derived from the AA and dry skull methods were used in MDA models, maximum incisor bite forces were underestimated in all three species relative to the extant models: by 38.8% in the squirrel, 21.8% in the guinea pig and 57.6% in the rat by the AA method, and by 76.7%, 64.5% and 51% by the dry skull method (figure 2*a,b*; electronic supplementary material, table S5). These errors meant that the AA iterations correctly identified the squirrel as having the highest bite force of the three morphotypes but misclassified the guinea pig and rat relative to each other. The dry skull method predicts the squirrel as having the lowest bite force rather than the highest but did correctly classify the rat as having a higher bite force than the guinea pig (figure 2*a,b*; electronic supplementary material, table S5).

The AA and dry skull model iterations differ in the nature and magnitude of error they yield in predictions of the mechanical efficiency of incisor biting across the rodent morphotypes (figure 2*c,d*; electronic supplementary material, tables S6 and S7). The AA model iterations underestimated mechanical efficiency in the rat and squirrel by 11% and 21.7% but overestimated it by 7.6% in the guinea pig (figure 2*c,d*; electronic supplementary material, tables S6 and S7). The dry skull method underestimated mechanical efficiency in all three taxa, by 15.3% in the rat, 23.9% in the squirrel and 25.6% in the guinea pig (figure 2*d*; electronic supplementary material, tables S6 and S7). Despite this error, the dry skull method did maintain the correct qualitative differences between the three morphotypes seen in the extant model iterations, with similarly high values of mechanical efficiency in the rat and squirrel and lower efficiency in the guinea pig (figure 2*c*; electronic supplementary material, table S6). However, the disparate nature of error in the AA model predictions resulted in this iteration incorrectly identifying the squirrel with the lowest mechanical efficiency (figure 2*c*; electronic supplementary material, table S6).

### 3.3*.* Bone stress

**Figure 3.**
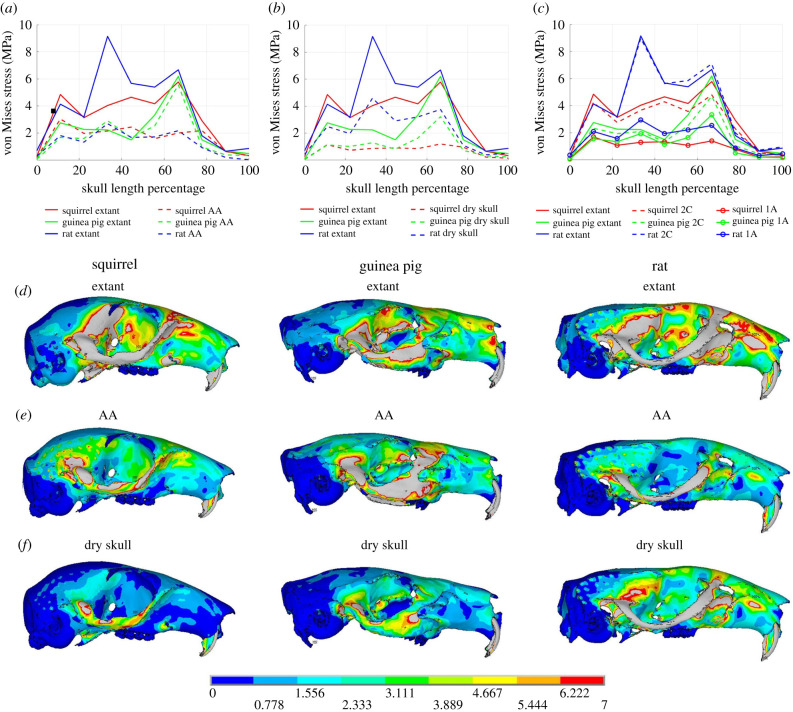
Comparison of stress magnitudes and distributions (represented by von Mises stress) along the length of the skull in the FE model iterations loaded using muscle properties measured in the rodent specimens being modelled (the extant model iterations) to model iterations where muscle properties were derived from (*a*) the AA method, (*b*) dry skull method and (*c*) muscle volume sculpture. In (*c*), only the most (2C) and least (1A) accurate iterations of the volume sculpture models from [36] are shown for comparative purposes. (*d–f*) Visualization of von Mises stress contour plots on the skulls themselves highlights the error in relative and absolute stress predicted in the (*e*) AA and (*f*) dry skull models versus the (*d*) extant model iterations, particularly along the zygomatic arch.

Here, we focus on stress outputs from FE models (figure 3) because tissue material properties in our models were set to standardized generic and homogeneous properties, mimicking the standard approach in macroevolutionary studies [36]. For completeness, strain outputs across model iterations are compared in the electronic supplementary material. FE models loaded with muscle forces derived from the MAA methods failed to capture many of the qualitative and quantitative patterns in bone stress observed in the extant model iterations (figure 3). With the exception of the guinea pig AA model (figure 3*a,e*), all MAA model iterations underestimate stress throughout the skulls: many require an increase of approximately 50% to reach the stress magnitudes in the extant iterations, while the worse performing models, such as the rat AA iteration (figure 3*a,e*), require more than a 400% to match the equivalent extant iteration. These large error magnitudes mean that both the AA and dry skull models fail to correctly order the rodent macroevolutionary morphotypes in their relative stress magnitudes. For example, the AA models suggest the rat experiences the lowest stress of the three morphotypes instead of the highest, while the guinea pig is (at certain points along the skull) recovered as experiencing the highest stresses rather than the lowest (figure 3*a,d,e*). The dry skull method also fails to recover the higher stresses expected in the squirrel versus guinea pig skull across most of skull length (figure 3*b,e,f*). Both MAA model types mostly capture the gross qualitative changes in stress along skull length in the rat and guinea pig models (e.g. higher stresses in the central skull length region associated with zygomatic arch). However, even gross changes in stress distribution are poorly captured in the squirrel, particularly in the dry skull iteration where the mean regional stress remains consistently low across skull length (figure 3).

## 4. Discussion and conclusion

MAA-based approaches to estimate muscle size and force-generating capacity, and subsequently bone loading, have been widely applied to extinct and extant taxa to examine the functional consequences of changing morphology and macroevolutionary patterns in the locomotor, axial and masticatory systems of vertebrates (e.g. [1–27]). Our study of its application to rodent masticatory morphotypes builds upon a small number of previous evaluations of such approaches [1,29–35] in a number of ways: by extending assessment to FE models; by providing assessment of qualitative and quantitative accuracy in an explicit macroevolutionary context; and by direct comparison to the most widely used alternative method of numerical soft tissue reconstruction (volume sculpture; e.g. [36–42]).

Previous studies that have examined the accuracy of the dry skull method have suggested that the approach overestimates the PCSA of the masseter muscles and medial pterygoid, while underestimating the PCSA of the temporalis [1,29–31]. Here, we find a different pattern of error, possibly owing to our taxonomic focus on rodents compared to that of previous evaluations of the dry skull method, which used opossums, carnivorans and bats. In this analysis, the masseter + medial pterygoid was underestimated by considerable amounts in all three rodent morphotypes, and the temporalis PCSA was considerably overestimated in the squirrel, underestimated in the rat, but accurately predicted in the guinea pig (figure 1*b*).

We also recover a complex pattern of error at the individual muscle level in our AA-based estimates (figure 1; electronic supplementary material, tables S1–S3). This approach underestimates PCSA in the superficial masseter, posterior deep masseter and medial and lateral pterygoids and overestimates temporalis PCSA in all three rodent morphotypes (figure 1; electronic supplementary material, tables S1–S3). However, the magnitude of this error varies enormously across the three species (figure 1*a*; electronic supplementary material, tables S1–S3). Like the dry skull method, other muscles show qualitatively variable error in the AA analysis across the three morphotypes; the anterior deep masseter PCSA is underestimated in the rat but overestimated in the squirrel and guinea pig. The infraorbital and posterior zygomatico-mandibularis muscles also show qualitatively different error across the studied taxa (figure 1; electronic supplementary material, tables S1–S3). Our relatively large errors in predicted PCSAs are qualitatively consistent with single taxon assessments of AA methods in humans [31,32] and macaques [33,34]. These studies recovered weak, and in some instances statistically insignificant, correlations between jaw muscle PCSA and a range of linear and area osteological attachment proxies and concluded that predictive relationships had considerable error margins [31–34]. However, these studies did not investigate the consequences of such error margins for functional metrics like bite force or bone loading.

Our findings highlight that the size of a muscle accommodation within or AA on the cranium is not necessarily a reliable guide to muscle PCSA, and that MAA-based approaches cannot necessarily be relied upon to produce systematic quantitative or even qualitative error across homologous muscles in different species (figure 1). This is further reflected in the relatively low frequency with which they correctly order the relative PCSAs of homologous muscles across the rodent morphotypes (the AA approach 10 out of 25 times; the dry skull method 1 out of 6 times). This level of relative accuracy given by the AA method lies towards the lower end of the range that Broyde *et al*. [36] recovered in these same three rodent specimens using muscle volume sculpture reconstruction. Using volume sculpture, one investigator recovered 29% accuracy in the relative ordering of muscle PCSA in these rodents, while two other investigators independently yielded 63% and 75% accuracy [36].

Sensitivity or parameter-specific error tests are relatively commonplace in both MDA and FE modelling studies (e.g. [38,39,41,42,48–56]). These studies provide a fundamental basis for understanding the absolute and relative impact of individual parameters on model predictions, thereby indicating which anatomical and physiological input variables must be most appropriately defined to ensure maximal model accuracy. Our anatomical reconstructions (figure 1) provide a new basis to examine the sensitivity of bite force and bone loading predictions specifically associated with MAA methods and macroevolutionary hypothesis testing (figures 2 and 3). Our MAA-based MDA models underestimated bite force in all three rodent morphotypes (figure 2*a,b*), which is qualitatively similar to the findings of previous evaluations of the dry skull method [1,29,30], except Davis *et al*. [31] who concluded that this approach accurately estimated bite forces in bats despite inaccurately predicting muscle parameters. However, the magnitude of underestimation varied considerably between rodent taxa (figure 2*a,b*). The AA models incorrectly predicted a higher incisor bite force in the guinea pig than the rat, while the dry skull method predicted the lowest bite force for the squirrel instead of the highest (figure 2*a,b*). These quantitative and qualitative errors warn against simply applying uniform correction factors or elevated values for maximum isometric stress to compensate for potential underestimation of bite force by MAA-based approaches [2,3,6,21].

Given mechanical efficiency is defined as the ratio between bite force and one of its major determinants, summed muscle force, it might be expected that this parameter would show very minor sensitivity to errors in PCSA (figure 1). In some model iterations, this does indeed appear to be the case (figure 2). However, larger errors in mechanical efficiency (greater than 20%) are seen where relatively large PCSA errors are focused in muscles with particularly small or large moments arms, such as the AA iteration of the squirrel model (figure 2; electronic supplementary material, tables S5–S7). Furthermore, this means that absolute or even relative error in mechanical efficiency is not predictable from error in PCSA or bite force alone: the summed muscle force and bite force are lower in AA model of the guinea pig than the extant model (figure 2*a,b*) iteration, yet mechanical efficiency is recovered as slightly higher in the AA iteration (figure 2*c,d*). Mechanical efficiency is considered a crucial functional adaptation that distinguishes sciuromorph, hystricomorph and myomorph rodents: squirrels (sciuromorph morphotype) are considered more efficient at muscle–bite force transmission during incisor gnawing than guinea pigs (hystricomorph morphotype), which matches the known diet of nuts and seeds that squirrels gnaw, and of grasses that guinea pigs grind down with their molars [46] (figure 2*c*). Rats (myomorph morphotype) are considered high performance generalists due to their high mechanical efficiency in both incisor and molar biting [46] (figure 2*c*). Because mechanical efficiency is similarly underestimated in all taxa, the dry skull method recovers the qualitative adaptive pattern correctly, although the distinction between squirrel and the rat is somewhat exaggerated relative to the extant model iteration (figure 2*c*). However, the AA method fails to recover this fundamental macroevolutionary signal: the squirrel is recovered with the lowest efficiency in incisor biting (figure 2*c*) and thus would be incorrectly interpreted as lacking the aforementioned adaptation for incisor gnawing of hard food types [46]. This might subsequently result in erroneous interpretations of the selective pressures driving the radiation of rodent macroevolutionary morphotypes. The majority of volume sculpture models of Broyde *et al*. [36] perform qualitatively and quantitatively better than MAA methods in mechanical efficiency (figure 2*c,d*). However, the potential for the same erroneous interpretation of inefficient incisor biting in the squirrel is also evident in the volume sculpture models of investigator 3 (VS—3a–3c; figure 2*c*).

To our knowledge, this study is the first to directly assess the accuracy with which MAA-based approaches produce quantitative and qualitative patterns of bone stress in FE models across a macroevolutionary radiation (figure 3). Our results demonstrate that even the most basic or gross pattern of stress distribution typically observed in mammalian skulls (considerably higher stress in the central skull regions in the zygomatic arch due to the attachment of large muscles to this relatively slender rod-like process) may not be recovered by FE models loaded with MAA-based muscle forces (figure 3*a,c,d*). While gross qualitative changes in stress along skull length are captured reasonably well in the rat and guinea pig models, relative patterns are more poorly captured in the squirrel models where stress remains much more uniform (figure 3). MAA-based models also fail to recover major qualitative differences between the morphotypes. For example, these models predict that the rat experiences the lowest stresses (instead of the highest) of the three species and fail to recover stress differences in zygomatic arch and posterior portion of the skull seen in models loaded with measured muscle data presented by Broyde *et al*. [36] and Cox *et al*. [46,47]. Recovery of highest stresses in the rat and lowest stresses in the guinea pig when models are loaded with measured muscle data are consistent with osteological and muscular differences between the myomorph and hystricomorph conditions. Rats (myomorph) have a large muscle mass to skull volume ratio, particularly in the zygomatic arch, orbital wall and temporal regions where the relatively large temporalis muscle of the rat generates higher stresses than are seen in the squirrel and guinea pig skulls [36,46,47] (figure 3*d*). By contrast, guinea pigs (hystricomorph) have relatively low overall muscle mass for their skull size, but also possess a more robust morphology of the zygomatic arch leading to lower stresses [36,46,47] (figure 3*d*). The failure to capture these qualitative adaptive differences, and indeed, the relatively poor performance of the MAA-based models overall, is a stark contrast to the accuracy of the volume sculpture model iterations presented by Broyde *et al*. [36], where the majority of models produced qualitatively accurate stress predictions and some iterations yielded extremely accurate quantitative predictions (figure 3*c*; electronic supplementary material, figure S8). Indeed, even the worst qualitatively performing volume sculpture model out-performs the MAA-based models presented here (figure 3*c*; electronic supplementary material, figure S8).

Herein, we have evaluated the quantitative and qualitative accuracy of MAA approaches relative to other biomechanical models (figures 2 and 3) in which nearly all muscle parameters were measured directly from the cadaveric specimens being modelled [43,46,47]. Given the relatively simple anatomical and functional activity under study (static maximal biting), it is likely that our ‘extant model’ iterations represent good approximations of reality and suitable benchmarks against which to measure the performance of MAA-based approaches in the context of macroevolutionary research. However, the use of a model (even one predominantly composed of species-specific input data) as a benchmark for other models would clearly be less appropriate in other circumstances. These might include, for example, more morphologically and functional complex situations (e.g. predictive whole-body simulations of locomotion with multiple bodies, linked by joints with higher degrees of freedom, controlled by large numbers of uni- and bi-articular muscles and interaction of several contact bodies with an environment). However, given our focus on static maximal incisor biting and the level of specimen-specific input data in our extant model iterations, we feel it is extremely unlikely that our quantitative and qualitative conclusions about the accuracy of MAA approaches would be altered by comparison to experimental data.

The extent to which the magnitudes of quantitative and qualitative error recovered here (figures 1–3) limit the predictive capability of MAA approaches is likely to vary according to the taxa and hypotheses under study. However, these results strongly suggest that MAA-based approaches are unlikely to accurately reproduce macroevolutionary changes in muscle proportions or biomechanical performance with high fidelity. Perhaps with the exception of mechanical efficiency (figure 2*c,d*), quantitative errors are consistently high and qualitative error is commonplace, resulting in the loss of anatomically and functionally defining features within individual species and erroneous conclusions about relative adaptations across rodent macroevolutionary morphotypes. It is currently rare for analyses of anatomical and functional evolution using MAA methods to formally acknowledge error in their hypothesis testing. Our results provide clear evidence of the need for this to become standard practice in order to objectively test or demonstrate the predictive capability of MAA-based estimates in the context of the functional and macroevolutionary hypotheses they have been constructed to test. In palaeontological studies, high levels of quantitative error may always persist due to need to reconstructively estimate most, if not all, force-generating muscle properties. However, error testing on extant taxa and the application of the resulting error margins to predictions of extinct taxa provides at least indirect evaluation of the predictive capabilities of models and their ability to provide meaningful tests of functional hypotheses [36,41,42,48,57]. Such studies also help to identify which parameters currently limit the predictive capabilities of models, and thus where future research investment in generating new methods and data might be best focused. The magnitudes of quantitative error and frequency of qualitative or relative error across models seen here (figures 1–3) suggest that current MAA methods do not represent a legitimate means to achieve the 3Rs in biomechanical studies of extant taxa. While a universal benchmark for model accuracy does not exist, it could be argued that near unanimous success in predicting relative or qualitative anatomical and functional differences between species or morphotypes represents a minimum threshold for a modelling method to serve as a valid alternative to direct experimentation on animals. If such were achieved, modelling approaches could be used instead of experimentation to test certain hypotheses about relative differences between species and qualitative cause–effect relationships in their functional anatomy. Unfortunately, our results suggest that MAA methods may, at present, fall short of that benchmark.

It seems clear that the failing of current MAA-based approaches comes from the assumption of a one-to-one relationship between AA and PCSA in each muscle, which is clearly not the case (figure 1). An alternative, and perhaps predictively superior approach, would be to examine the scaling relationship between MAA and gross properties (volume, PCSA) within homologous muscles across a large number of species. Similar approaches are widely used for estimating body mass based on various skeletal proportions (e.g. [58,59]) and have the advantage of delivering statistically based estimates with confidence intervals that permit objective and systematic error testing in subsequent biomechanical models [36,41,42,48,49,57]. We, therefore, suggest that future work should go back to the bones to test if large datasets can yield strong predictive relationships between MAAs and muscle properties (volume, PCSA) for use in macroevolutionary and functional studies.

## Data accessibility

All models are available from the Dryad Digital Repository: https://doi.org/10.5061/dryad.kd51c5b4c and the University of Liverpool's DataCat facility: https://doi.org/10.17638/datacat.liverpool.ac.uk/1184.
